# Relationship between HIF-1 and Circadian Clock Proteins in Obstructive Sleep Apnea Patients—Preliminary Study

**DOI:** 10.3390/jcm9051599

**Published:** 2020-05-25

**Authors:** Agata Gabryelska, Marcin Sochal, Szymon Turkiewicz, Piotr Białasiewicz

**Affiliations:** Department of Sleep Medicine and Metabolic Disorders, Medical University of Lodz, 90-419 Lodz, Poland; marcin.sochal@umed.lodz.pl (M.S.); szymon.turkiewicz@stud.umed.lodz.pl (S.T.); piotr.bialasiewicz@umed.lodz.pl (P.B.)

**Keywords:** obstructive sleep apnea (OSA), polysomnography (PSG), hypoxia, hypoxia-inducible factor (HIF), circadian rhythm, circadian clock proteins

## Abstract

Obstructive sleep apnea (OSA) is characterized by intermittent hypoxia and associated with the disruption of circadian rhythm. The study aimed to assess the relationship between hypoxia-inducible factor (HIF) subunits, circadian clock proteins, and polysomnography (PSG) variables, in healthy individuals and severe OSA patients. The study included 20 individuals, who underwent PSG and were divided into severe OSA group (*n* = 10; AHI ≥ 30) and healthy control (*n* = 10; AHI < 5) based on apnea-hypopnea index (AHI). All participants had their peripheral blood collected in the evening before and the morning after the PSG. HIF-1α, HIF-1β, BMAL1, CLOCK, CRY1, and PER1 protein concertation measurements were performed using ELISA. In a multivariate general linear model with the concentration of all circadian clock proteins as dependent variables, evening HIF-1α protein level was the only significant covariant (*p* = 0.025). Corrected models were significant for morning and evening PER1 (*p* = 0.008 and *p* = 0.006, respectively), evening (*p* = 0.043), and evening BMAL protein level (*p* = 0.046). In corrected models, evening HIF-1α protein level had an influence only on the evening PER1 protein level. Results suggest that OSA patients are at risk for developing circadian clock disruption. This process might be mediated by subunit α of HIF-1, as its increased protein level is associated with overexpression of circadian clock proteins.

## 1. Introduction

Obstructive sleep apnea syndrome (OSA) is a chronic condition characterized by recurrent pauses in breathing during sleep, which leads to intermittent hypoxia (IH), hypercapnia, arousals, and sleep fragmentation. Recent studies suggest that the prevalence of sleep disordered breathing drastically increased and in moderate or severe form might affect up to 50% men and 24% women [1]. 

One of the typical complications of sleep disordered breathing is recurrent hypoxia, which subsequently causes modifications of gene expression. The main factor responsible for oxygen metabolism homeostasis is hypoxia-inducible factor 1 (HIF-1). It is a heterodimeric complex, which consists of two subunits: α (HIF-1α) and β (HIF-1β). Both subunits belong to helix-loop-helix (bHLH)—Per/Armt/Sim (PAS) transcription factor family [2]. Subunit α is oxygen-sensitive, while the stability of the β subunit does not depend on oxygen partial pressure [2]. Recent studies have shown that HIF-1α serum protein level is chronically increased in OSA patients and is not reverted by one-night continuous positive air pressure therapy [3,4].

Circadian clocks are endogenous coordinators of the 24-h rhythm of behavioral and molecular processes in living organisms. For humans, a master clock modulating circadian rhythms is located in the suprachiasmatic nucleus of the hypothalamus and is a pacemaker of a hierarchical system [5]. In mammals, the circadian clock is comprised of a set of genes, which function as activators—CLOCK and BMAL, which, similarly to HIF, are bHLH-PAS transcription factors [5]. Through binding to regulatory elements containing E-boxes (also present in HIF-1α gene) they activate the transcription of repressor protein period (PER) and cryptochrome (CRY). Additionally, HIF can bind to promoter regions of repressor proteins through hypoxia response elements (HRE), causing their upregulation [5]. Literature concerning circadian clock disruption among patients suffering from OSA is limited. In a study that compared eight healthy individuals and eight patients suffering from OSA, Burioka et al. found disruption of mRNA expression level of *PER1* and showed that 3 months CPAP treatment reversed it [6]. Moreira et al. found that only expression of *CLOCK* mRNA level was altered in 13 OSA patients, compared to eight healthy individuals, and was not affected by 1 month CPAP therapy [7].

To the best of our knowledge, there is no literature regarding the relationship between hypoxia and the circadian clock in OSA patients. The study aimed to investigate differences in concentration of chosen circadian clock proteins in OSA patients and healthy participants and evaluate the association between them and HIF-1 protein subunits as well as selected polysomnography (PSG) parameters.

## 2. Materials and Methods

### 2.1. Sample

The study group included 20 patients, who were referred to Sleep and Respiratory Disorders Centre in Lodz (Poland) with a presumptive OSA diagnosis and underwent standard nocturnal PSG examination (Jaeger, Viasys Healthcare, Höchberg, Germany), which was scored according to American Academy of Sleep Medicine guidelines [8]. PSG examination room had a set temperature at 20 °C. Time of examination was 9 h (lights out: 21:00–21:30; lights on: 6:00–6:30). Based on apnea-hypopnea index (AHI), patients were divided into severe OSA group (*n* = 10; AHI ≥ 30; 90% male) and healthy control (*n* = 10; AHI < 5; 70% male). Patients diagnosed with any chronic respiratory conditions (e.g., bronchial asthma, or chronic obstructive pulmonary disease) and any sleep disorders other than OSA (e.g., insomnia, delayed phase syndrome) were excluded from the study. Additionally, exclusion criteria included chronic inflammatory diseases (e.g., connective tissue diseases or inflammatory bowel diseases), any infection within one month of blood collection, diagnosis of cancer (active or in medical history), psychiatric disorders and shift work system, and jet lag due to a flight within 2 weeks of the study or taking medications affecting sleep (e.g., benzodiazepines and melatonin). Participants followed a feeding schedule, with the last meal of the day being consumed at 18:00. Participants were asked to restrain from naps during the daytime. The study was approved by the Ethical Committee of Medical University of Lodz (RNN/432/18/KE); all patients provided written informed consent to participate in the study.

### 2.2. Material Collection and Protein Level Assessment

Peripheral blood samples were collected in the evening (at 21:00–21:30, 15 min before lights out)—before and in the morning (at 6:00–6:30, 15 min after light on)—following PSG examination, and centrifuged. Serum was collected and stored at −80 °C. Enzyme-linked Immune Sorbent Assay kit was used to assess serum protein concentrations: HIF-1α (detection range (DR): 81.92–20,000.00 pg/mL; sensitivity (S) <30 pg/mL; dilution (D): none) (Invitrogen, Carlsbad, CA, USA), HIF-1β (DR: 0.15–10.00 ng/mL; S < 0.056 ng/mL; D: 1:20), CLOCK (DR: 0.15–10.00 ng/mL; S < 0.089 ng/mL; D: 1:10), BMAL1 (DR: 78.00–5000.00 pg/mL; S < 38.0 pg/mL; D: 1:50), PER1 (DR: 1.56–100.00 ng/mL; S < 0.089 ng/mL; D: 1:10) (EIAab, Wuhan, China), and CRY1 (DR: 0.313–20.00 ng/mL; S < 0.188 ng/mL; D: 1:10) (FineTest, Wuhan, China).

### 2.3. Statistical Analysis

Statistical analysis was performed with SPSS 25.0 (IBM, Chicago, IL, USA). Normal distribution was evaluated by the Shapiro–Wilk test. Comparisons between the groups for data with normal distribution were performed by independent-samples *t*-test and results presented as mean ± standard deviation, while for data without normal distribution Mann–Whitney U test was used and results presented as median and interquartile range. Chi^2^ test was used for categorical variables. For repeated dependent measurements (evening-morning protein concentration differences), paired samples *t*-test was used for data with normal distribution and Wilcoxon test for data with non-normal distribution. The multivariate general linear model was performed to analyze the influence of OSA severity (AHI), desaturation index (ODI), evening HIF-1α protein level, and BMI on the concentration of all circadian clock proteins. Correlations for circadian clock proteins with HIF-1α protein level in the evening with *p* < 0.01 in corrected models were assessed by the Spearman test; the significance of multiple correlations were manually adjusted by the Holm-Bonferroni method. The level of significance was set at *p* < 0.05.

## 3. Results

Characterization of both study groups including demographic and PSG parameters, as well as protein concentrations, are shown in Table 1. No differences in the evening and morning protein concentration were observed in either group (*p* > 0.05), except for PER1 and CRY1 (both *p* < 0.001).

**Figure 1:**
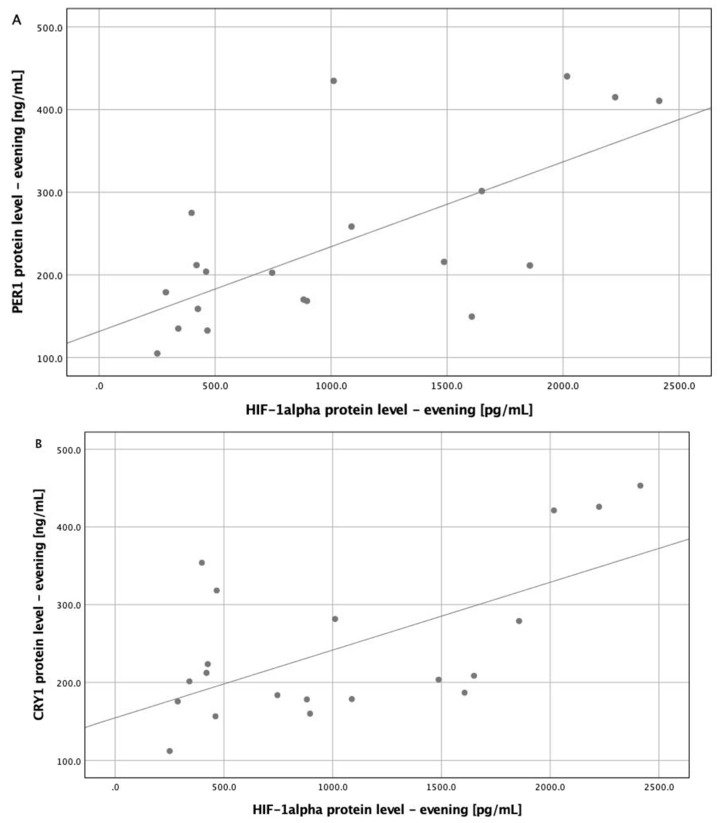

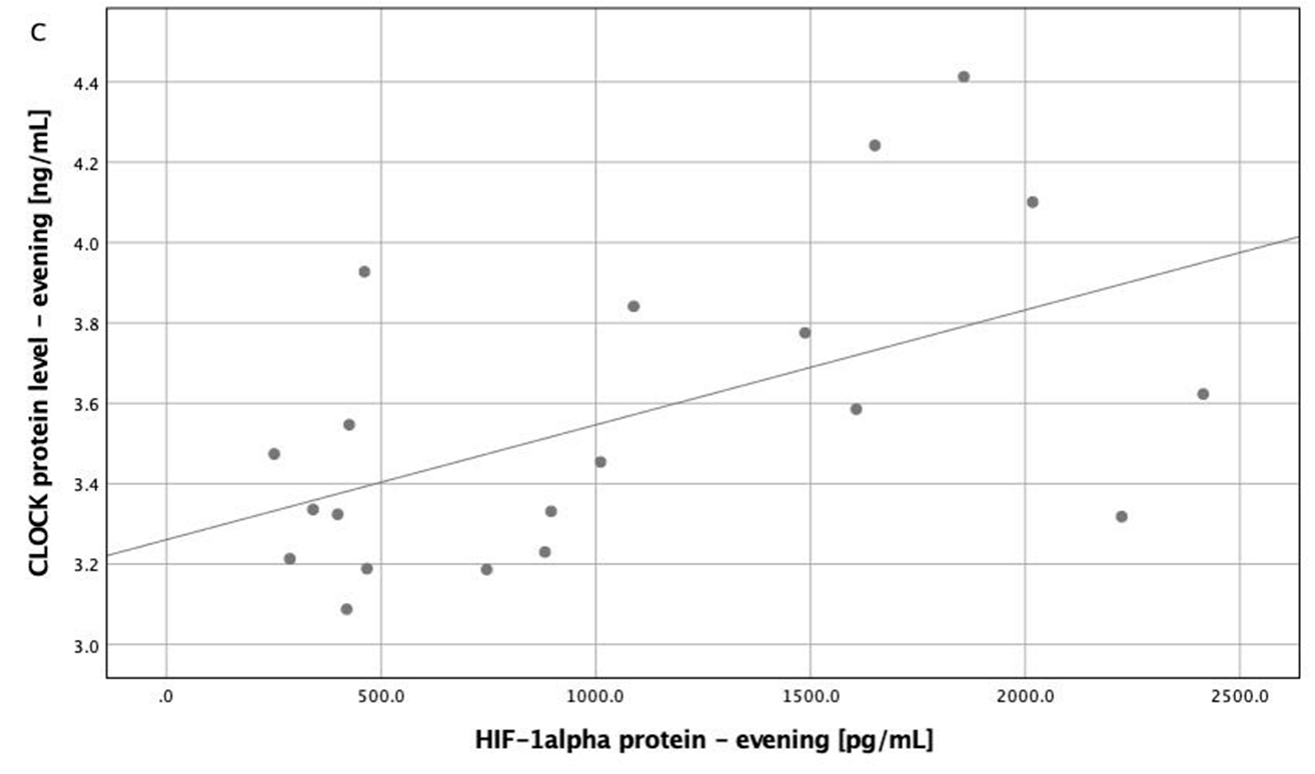
Correlations between evening HIF-1α protein level and circadian proteins (PER1, CRY1, and CLOCK). (**A**) Correlation between evening hypoxia-inducible factor (HIF) 1α protein level and evening period 1 (PER1) protein level (*R* = 0.618, *p* = 0.004). (**B**) Correlation between evening hypoxia-inducible factor (HIF) 1α protein level and evening cryptochrome 1 (CRY1) protein level (*R* = 0.514, *p* = 0.020). (**C**) Correlation between evening hypoxia-inducible factor (HIF) 1α protein level and evening Circadian Locomoter Output Cycles Protein Kaput (CLOCK) protein level (*R* = 0.618, *p* = 0.004).

In the multivariate general linear model with the concentration of all circadian clock proteins as dependent variables, evening HIF-1α protein level was the only significant covariant (*p* = 0.025). Corrected models were significant for morning and evening PER1 protein levels (*p* = 0.008 and *p* = 0.006, respectively), evening CRY1 protein level (*p* = 0.043), and evening BMAL protein level (*p* = 0.046). In corrected models, evening HIF-1α protein level had an influence on the evening PER1 protein level (*p* = 0.020) (Table 2). Positive correlations between evening PER1, CRY1, CLOCK proteins, and evening HIF-1α protein level were found (*R* = 0.618, *p* = 0.004; *R* = 0.514, *p* = 0.020 and *R* = 0.511, and *p* = 0.021 respectively) (Figure 1).

## 4. Discussion

Intermittent hypoxia is the main contributor of OSA-related pathologies, putting the patients at risk of developing multiple comorbidities, particularly cardiovascular and metabolic disorders [9]. In this preliminary study, we demonstrate that levels of circadian clock proteins in peripheral blood are increased in OSA compared to healthy individuals, which might suggest that they are likely to suffer from dysregulation of the circadian rhythm. This might be possibly mediated by HIF-1.

Several studies have investigated the changes in mRNA expression of circadian clock genes, but not investigated them on protein level. One of the studies observed disruption of the *Per1* expression profile, which was reverted and similar to healthy individuals following 3 months of CPAP treatment [6]. However, Moreira et al. found that only expression of *CLOCK* (but not *BMAL*, *PER,* and *CRY*) was increased in OSA patients and did not decrease following one month of CPAP treatment [7]. Another study showed that *BMAL1*, *CLOCK,* and *CRY1* were arrhythmic in patients with OSA, with *BMAL1* expression being increased in the morning in the OSA group compared to healthy control [10]. Canales et al. investigated a panel of circadian clock genes in veterans with chronic kidney disease, and patients with OSA had decreased only *PER3* mRNA expression [11]. In the same study, groups were created based on the presence of nocturnal hypoxemia (NH) defined as ≥10% of total sleep time spent below 90% oxygen saturation, and individuals with NH had decreased expression of *PER1* and *Rev-erbα* [11]. Yet, it is hard to determine if OSA per se is a decisive factor in the downregulated expression of circadian clock genes in this group, as it has been shown that kidney function influences their expression [12]. Compared to the aforementioned results, our study shows an overwhelming increase in circadian clock proteins level, both in the morning and in the evening. This might be caused by possible additional control of the translational process, which might result in an intensification of observed differences on the protein level. Nevertheless, verification of this result in a larger group and a comparison of expression on both protein and mRNA levels in the same individuals is needed.

To the best of our knowledge, this is the first study to evaluate the association between HIF-1 subunits and circadian clock protein levels in OSA patients. This relationship is particularly important, as OSA individuals have increased HIF-1α protein levels [3]. Adamovich et al. found that in cultured cells, physiological oxygen rhythms synchronize clock genes in HIF-1α-dependent manner. Additionally, in the same study it has been shown that modulation of oxygen levels quickens the recovery of wild-type mice from a jet lag protocol, but not that of HIF-1α-deficient mice [13], while Manella et al. have shown a misalignment of circadian clock in mice model of OSA [14]. Furthermore, mice exposed to hypoxia had increased levels of PER1 and CLOCK proteins [15]. The multivariate model generated in the study has shown that the protein level of HIF-1α significantly affected the concentration of circadian clock proteins, while disease severity, desaturation index, and BMI did not. This is in line with the aforementioned results from animal models. Moreover, in models corrected for each of the evaluated circadian proteins, the level of HIF-1α protein remained significant for PER1 in the evening. This might be possibly caused by the fact that this repressor protein has HRE in the promotor of the encoding gene, which allows for HIF binding, resulting in the expression of this protein [5]. Moreover, association with HIF-1α is only present in the evening measurements of the circadian clock repressor protein. This might be due to the accumulation of PER1 protein in the afternoon, which enhances the effect present only in the evening but not in the morning [5]. Similar results would be expected for the other repressor protein, CRY1; however, in the corrected model evening HIF-1α protein level did not reach a significant level, which might be caused by an insufficient number of participants. The small size of the group is the main limitation of the study, therefore the results obtained should be interpreted with caution.

The results of our study suggest that OSA patients are at risk of developing circadian clock disruption. This process might be mediated by the subunit α of HIF-1, as its increased level is associated with the overexpression of circadian clock proteins. Further studies, both in a larger cohort and with prospective observation of treatment effects, are necessary to fully understand this complex relationship.

## 5. Conclusions

Results of the study suggest that OSA patients are at risk for developing circadian clock disruption. This process might be mediated by subunit α of HIF-1, as its increased level is associated with overexpression of circadian clock proteins. Nevertheless, further research on larger population are needed to verify this complex relationship.

## Figures and Tables

**Table 1:** Comparison of the study groups.

	OSA Group (*n* = 10)	Control Group (*n* = 10)	*p* Value
Age (years)	50.30 ± 9.75	49.00 ± 10.19	0.774
BMI (kg/m^2^)	34.48 ± 2.95	28.1 ± 4.1	0.001
Sex	90%M	70%M	0.264
HIF-1α evening (pg/mL)	1287.64 (452.31–2069.13)	606.45 (327.97–1073.51)	0.020
HIF-1α morning (pg/mL)	1236.97 ± 871.44	742.06 ± 450.65	0.043
HIF-1β evening (ng/mL)	72.59 ± 0.94	71.50 ± 1.32	0.047
HIF-1β morning (ng/mL)	72.38 ± 1.08	71.26 ± 1.65	0.091
CLOCK evening (ng/mL)	3.73 ± 0.35	3.38 ± 0.33	0.037
CLOCK morning (ng/mL)	3.52 ± 0.15	3.31 ± 0.20	0.019
BMAL1 evening (ng/mL)	9.32 ± 0.51	9.00 ± 0.40	0.132
BMAL1 morning (ng/mL)	9.52 ± 0.47	8.87 ± 0.61	0.016
PER1 evening (ng/mL)	266.75 (209.54–419.87)	169.32 (134.50–204.97)	0.004
PER1 morning (ng/mL)	38.01 (30.05–68.96)	23.84 (18.22–38.39)	0.029
CRY1 evening (ng/mL)	280.26 (197.31–422.36)	185.21 (171.59–209.50)	0.035
CRY1 morning (ng/mL)	39.38 (30.55–43.43)	27.95 (15.97–40.84)	0.105
TST (h)	6.43 ± 0.83	6.08 ± 0.68	0.321
Percentage of time of TST spent in REM stage (%)	19.03 ± 7.01	20.85 ± 8.09	0.597
Arousal Index	24.80 (20.15–34.18)	15.20 (6.73–19.75)	0.005
AHI	56.40 (44.98–65.33)	1.30 (0.38–3.00)	<0.001
AHI REM	29.81 (17.11–56.67)	0.00 (0.00–4.88)	<0.001
AHI nREM	46.99 (33.54–64.91)	1.07 (0.20–1.42)	<0.001
ODI	57.85 (45.00–84.83)	2.00 (0.98–3.00)	<0.001
Awake SpO_2_	89.85 (83.65–93.60)	93.85 (92.45–94.85)	0.005
Mean SpO_2_ of desaturation	84.60 (74.50–89.73)	91.75 (90.35–92.90)	<0.001
Minimal SpO_2_	63.45 (40.85–75.18)	88.90 (84.73–91.90)	<0.001
Time spent with SpO_2_ below 90% (min)	107.95 (73.05–278.58)	0.55 (0.00–5.50)	<0.001
Percentage of time with SpO_2_ 90% (%)	27.96 (20.44–64.69)	0.15 (0.00–1.48)	<0.001

Variables with normal distribution is presented as mean ± SD; variables with a non-normal distribution are shown as median (IQR). AHI, apnea-hypopnea index; BMAL, Brain and Muscle Aryl Hydrocarbon Receptor Nuclear Translocator-Like 1; BMI, body mass index; CLOCK, Circadian Locomoter Output Cycles Protein Kaput; CRY, cryptochrome; HIF, hypoxia-inducible factor; ODI, Desaturation Index; PER, period; REM, rapid eye movement; SpO_2_, hemoglobin oxygen saturation; TST, total sleep time.

**Table 2:** Multivariate general linear model of circadian clock proteins.

		Corrected Model		HIF-1α Protein Level-Evening	AHI	ODI	BMI
		*p* Value	*R* Squared	*p* Value			
Protein Level	Multivariate General Linear Model	-		0.025	0.123	0.131	1.505
	CLOCK evening	0.046	0.455	0.056	0.064	0.074	0.699
	CLOCK morning	0.059	0.435	0.654	0.086	0.356	0.115
	BMAL1 evening	0.707	0.126	0.687	0.387	0.596	0.415
	BMAL1 morning	0.057	0.438	0.143	0.795	0.674	0.321
	PER1 evening	0.008	0.583	0.020	0.379	0.627	0.784
	PER1 morning	0.006	0.593	0.972	0.012	0.003	0.662
	CRY1 evening	0.043	0.461	0.052	0.903	0.706	0.707
	CRY1 morning	0.487	0.194	0.629	0.604	0.746	0.400

AHI, apnea-hypopnea index; BMAL, Brain and Muscle Aryl Hydrocarbon Receptor Nuclear Translocator-Like 1; BMI, body-mass index; CLOCK, Circadian Locomoter Output Cycles Protein Kaput; CRY, cryptochrome; HIF, hypoxia-inducible factor; PER, period.
